# Structural Neuroimaging in Substance-Induced Psychosis

**DOI:** 10.1007/s11920-026-01678-0

**Published:** 2026-04-30

**Authors:** Murad Atmaca, Muhammed Fatih Tabara

**Affiliations:** School of Medicine, Department of Psychiatry, Firat University, Firat Tip Merkezi, Psikiyatri Anabilim Dali, Elazig, 23200 Türkiye

**Keywords:** Substance-induced psychosis, Neuroimaging, Structural MRI, Cannabis, Methamphetamine

## Abstract

**Purpose of Review:**

This review evaluates structural neuroimaging research on substance-related psychosis to determine if these conditions share common neurobiological pathways with primary psychotic disorders, such as schizophrenia, or exhibit unique signatures.

**Recent Findings:**

Research indicates that methamphetamine, cannabis, and cocaine induce significant structural changes, including cortical thinning and grey matter loss. Methamphetamine-induced psychosis is associated with frontal lobe reductions and smaller hippocampal and amygdalar volumes. Cannabis use is linked to alterations in the cingulate cortex and cerebellar networks, particularly when use begins early in life. While many neuroimaging patterns overlap with schizophrenia substance-specific variations exist. For instance, methamphetamine users show more pronounced amygdalar reduction than those with primary psychosis. Generally, structural deficits are more severe and widespread in schizophrenia than in purely substance-induced cases.

**Summary:**

Substance-induced psychoses exhibit neuroanatomical signatures largely similar to primary psychoses, though distinct regional variations suggest partially divergent mechanisms. Future longitudinal and multimodal research is essential to identify definitive biomarkers for clinical differentiation.

## Introduction

Psychotic disorders, particularly schizophrenia, are severe psychiatric conditions in which the ability to evaluate reality is profoundly impaired. These disorders are defined as a cluster of serious psychiatric conditions characterised by abnormal perception and thought. Both negative symptoms, reflected in the reduction or absence of normal functions (e.g., alogia, avolition, and diminished self-care), and positive symptoms, reflected in the exaggeration of normal functions (e.g., delusions, hallucinations, and disorganised speech or behaviour), are typically observed.

In primary psychotic disorders, notably schizophrenia, structural neuroimaging reveals a consistent pattern of widespread grey and white matter alterations that serves as a vital benchmark for differentiating substance-induced states. The most robustly replicated findings include diminished total brain and grey matter volumes alongside the enlargement of the lateral and third ventricles, a feature often detectable at the first episode of psychosis. Furthermore, widespread reductions in cortical thickness are observed—particularly within the frontal and temporal lobes—while significant volume loss is frequently identified in the hippocampus, amygdala, and thalamus. Conversely, certain subcortical regions, such as the caudate nucleus, putamen, and nucleus accumbens, often exhibit increased volume; these hypertrophic changes are generally attributed to the secondary effects of long-term antipsychotic treatment. Finally, compromised white matter integrity, evidenced by reduced fractional anisotropy in tracts such as the corpus callosum, suggests a profound disruption in connectivity between distant cortical regions.

According to the Diagnostic and Statistical Manual of Mental Disorders, Fifth Edition (DSM-5), substance-induced psychosis is defined by the presence of delusions and/or hallucinations that emerge during or shortly after substance ingestion or acute withdrawal. A key clinical feature is that these symptoms are generally expected to remit following approximately one month of abstinence [1]. When symptom clusters extend beyond this duration, considerable difficulties are encountered in differentiating substance-related psychosis from schizophrenia and other primary psychotic disorders in clinical practice [2]. In a follow-up study, patients with primary psychosis and those with substance-induced psychosis admitted to a psychiatric unit were compared at baseline and at subsequent intervals until discharge. Patients with substance-induced psychosis recovered more rapidly than those with a primary psychotic disorder, with the difference being most pronounced during the first 15 days. Nevertheless, both groups exhibited comparable positive symptom severity, while more severe behavioural disturbances were observed among patients with substance-induced psychosis [3]. Contrary evidence has been reported in a study where patients with substance-induced psychosis did not exhibit a faster rate of remission following substance withdrawal compared to those with a primary psychotic disorder and co-occurring substance use. On the contrary, a slower reduction in hallucinations relative to baseline levels was observed [4].

The use of addictive substances has increasingly become a major public health concern worldwide. According to the European Monitoring Centre for Drugs and Drug Addiction, approximately 30% of adults in the European Union aged 15–64 have used illicit substances, particularly cannabis, at least once in their lifetime. Among young adults aged 15–34, considered the group most vulnerable to addiction, it has been estimated that 16% used cannabis within the past year, with prevalence reported to be approximately twice as high among men [5]. It has been estimated that approximately 4% of the global population, including both young adults and adults, have used cannabis at least once. Moreover, cannabis use has been reported to have increased by nearly 20% over the past decade [6]. An important consideration in substance-induced psychosis is the dose–response relationship in cannabis use, as the risk of psychosis has been shown to increase significantly with higher levels of tetrahydrocannabinol (THC) [7, 8]. A particularly alarming trend in public health is that the age of initiation for substance use is becoming progressively younger. In 2023, cannabis use within the past year was reported by 21.8% of individuals aged 12 years or older, corresponding to approximately 61.8 million people. Prevalence was highest among young adults aged 18–25 (36.5%, or 12.4 million), followed by adults aged 26 and older (20.8%, or 46.5 million), and adolescents aged 12–17 (11.2%, or 2.9 million) [9].

Cocaine, one of the most commonly used illicit substances, is estimated to affect nearly 25 million people worldwide, particularly in Americas, and Europe [10]. Among users, between half and three-quarters are reported to experience vivid hallucinations, delusions, and paranoid thoughts [11–13].

Methamphetamine use, increasingly encountered in psychiatric practice due to its availability and relatively low cost, is frequently associated with acute psychotic symptoms. In one study, nearly one-quarter of individuals using methamphetamine were reported to exhibit a psychotic disorder, indicating a markedly elevated risk and underscoring the severity of the problem [14].

Research on the prevalence of substance-related psychosis remains limited. In one study, the incidence of primary psychotic disorder was reported as 24.1 per 100,000 persons per year, while the incidence of primary psychotic disorder with comorbid substance misuse was 9.7, exceeding that of substance-related psychotic disorder, which was 6.5 [15]. With respect to treatment referral rates, substance-related psychosis has been reported to account for approximately 6–10% of patients presenting with first-episode psychosis compared with those presenting with primary psychotic or affective disorders [16, 17].

Distinguishing substance-induced psychosis from primary psychotic disorders remains a major clinical challenge. Although certain indicators exist, differentiation is often difficult, which likely affects research on substance-related psychosis, including structural neuroimaging studies reviewed here. It should also be considered that individuals predisposed to primary psychotic disorders may be more likely to use substances, while conversely, substance use may accelerate the course of an underlying psychotic condition [18]. Notably, substance-induced psychosis may occur at older ages and outside the typical onset range expected for primary psychotic disorders, further complicating diagnosis [17, 19]. Furthermore, individuals with substance-induced psychosis have been reported to show greater insight into their psychotic symptoms—particularly delusions and hallucinations—and to exhibit fewer negative symptoms compared with those with primary psychotic disorders [17, 19]. Regarding positive symptoms, some studies have reported higher levels in substance-induced psychosis, whereas others have found no such difference [15, 17, 19, 20].

Although primary psychotic disorders and substance-induced psychoses differ in certain respects, a diagnosis of schizophrenia or bipolar disorder is often made in the long term following substance use. For instance, approximately one-third of individuals diagnosed with amphetamine-induced psychosis are subsequently diagnosed with schizophrenia or bipolar disorder. Conversely, this proportion is reduced to approximately one-quarter among those diagnosed with hallucinogen-induced psychosis. Cannabis appears to play the most prominent role, with nearly half of those experiencing cannabis-induced psychosis later diagnosed with schizophrenia [21].

Extensive efforts have been made to explain the diverse clinical features of primary psychotic disorders, particularly schizophrenia, through structural and functional neuroimaging. However, neuroimaging studies on psychotic states arising from substance use disorders remain limited. This review therefore aims to summarise the available structural neuroimaging research and provide insights for future work in this field.

## Methods

The review was designed in accordance with the PRISMA guidelines. In selecting studies for this review, publications directly or indirectly related to the topic were identified in Turkish, English, and French across major databases, including Embase, PsycINFO, MEDLINE, Google Scholar, Cochrane Library, Web of Knowledge, CINAHL, and LILACS. After a preliminary screening, relevant studies were included. In order to maintain a focused and scientifically rigorous analysis, the following eligibility criteria were applied:

### Inclusion Criteria

- Study Type: Only original research articles involving structural neuroimaging techniques (e.g., MRI, CT).
- Participant Population: Patients diagnosed with substance-induced psychosis.
- Search Scope: While the search strategy included terms for cannabis, stimulants (methamphetamine, cocaine), hallucinogens (LSD, psilocybin), ecstasy (MDMA), opioids, these were included in the criteria to ensure a comprehensive review of illicit drug-induced states.
- Comparative Scope: Studies comparing substance-induced psychosis to healthy controls, non-psychotic substance users, or patients with primary psychotic disorders (e.g., schizophrenia).
- Language: Studies published in Turkish, English, or French.
- Timeframe: Publications from 1980 to July 2025.

### Exclusion Criteria

- Modality: Purely functional neuroimaging studies (e.g., fMRI, PET) unless they also included structural data.
- Content: Case reports, editorials, or conference abstracts that did not provide primary structural data.
- Substance type (alcohol): Studies focusing primarily on alcohol-induced psychotic disorders were excluded. This is because chronic alcohol use is associated with extensive, widespread structural deficits (e.g. atrophy) that can make it difficult to distinguish the specific neuroanatomical markers of the psychotic state from general alcohol-related neurodegeneration.

To ensure the rigour of the selection process, both authors (M.A. and M.F.T.) independently screened the titles and abstracts of all identified records. Full-text articles were then reviewed by both authors to determine final eligibility based on the predefined inclusion and exclusion criteria. Any disagreements regarding the inclusion of specific studies were resolved through collaborative discussion until a consensus was reached. This dual-reviewer approach was utilised to minimise selection bias and ensure a comprehensive evaluation of the structural neuroimaging literature.

Given the limited literature on this topic, a small number of studies examining the association between substance use and psychosis risk—though not conducted directly in patients with substance-induced psychosis—were also included. Some search words or phrases were selected to find relevant studies in the literature. These search terms can be found in the Appendix.

### Data Extraction

A standardised data extraction form was used to collect relevant information from each included study. Both authors (M.A. and M.F.T.) were involved in pulling data to ensure accuracy. The extracted data included:

- Study characteristics (e.g., author, year, country).
- Participant demographics (e.g., age, sex, primary substance used).
- Neuroimaging modality and analysis methods (e.g., MRI, voxel-based morphometry, cortical thickness).
- Key structural findings (e.g., regional volume reductions, cortical thinning).
- Clinical correlations (e.g., symptom severity, duration of abstinence).

### Quality and Bias Assessment

A qualitative assessment approach, based on common neuroimaging research standards, was utilised to assess the risk of bias and the methodological quality of the included studies. The included studies were specifically evaluated for:

- Selection Bias: Whether the study matched patient groups with healthy controls for age, sex, and intracranial volume.
- Confounding Factors: Whether the studies controlled for the use of other substances (e.g., alcohol, tobacco) and medication effects.
- Measurement Bias: The use of standardised and automated analysis software versus manual tracing.
- Sample Size and Power: Whether the sample sizes were sufficient to draw definitive conclusions.

## Results and Discussion

### Overview of Included Literature

Following the systematic selection process, a total of 14 primary research studies were identified that met the inclusion criteria for this review. The distribution of the literature reflects a growing but still limited evidence base compared to primary psychotic disorders. The identified studies are categorised as follows: six focusing on methamphetamine-induced psychosis, which include research on voxel-based morphometry, blood transcriptome analysis, cortical thickness, and medial temporal lobe structures. Six studies were classified under cannabis-induced psychosis, including research on long-term users, surface-based morphometry, and changes in the cingulate cortex. Additionally, three studies investigated related factors, such as genetic polymorphisms in heavy users, grey matter networks linked to the age of initiation, and subcortical volumes in individuals at clinical high risk for psychosis. Finally, two studies focused on cocaine- and stimulant-induced psychosis, comparing cocaine-dependent individuals with and without psychosis and examining regional brain volumes and white matter connectivity in stimulant-induced psychosis versus schizophrenia. While the number of studies is growing, the literature remains limited compared to primary psychotic disorders, highlighting the need for more specialised structural neuroimaging research in this field. A comprehensive overview is provided in Table 1.

**Table 1:** Comprehensive summary of the studies

Research	Sample size (Case / Control)	Neuroimaging method	Regions of interest	Results
Aoki et al. (2013)	20 Methamphetamine-associated psychosis (MAP) 20 Healthy controls (HCs)	Structural MRI	Superior temporal gyrus Frontopolar cortex (FPC) Ventrolateral FPC Ventromedial FPC Dorsomedial FPC Dorsolateral FPC Orbitofrontal area	MAP patients showed reduced grey matter in left perisylvian and frontopolar regions, and less white matter in the orbitofrontal area
Breen et al. (2016)	10 Non-psychotic methamphetamine users (NPMU) 10 MAP 10 HCs	Structural MRI	Hippocampus Caudate Nucleus accumbens Putamen Ventral diencephalon Corpus callosum	Significantly lower bilateral hippocampal volumes in MAP subjects
Uhlmann et al. (2016)	21 NPMU 19 MAP 19 HCs	Structural MRI	Fusiform Inferior temporal gyrus (ITG) Orbitofrontal cortex (OFC) Inferior frontal gyrus (IFG) Insula Anterior cingulate cortex Temporal cortex Hippocampus	Thinner fusiform, ITG, OFC, IFG, and insula in MAP group Lower hippocampal volumes in MAP group
Orikabe et al. (2011)	20 MAP 20 HCs	Structural MRI	Total grey matter Hippocampus Amygdala	MAP subjects: smaller bilateral amygdala and hippocampus volumes than controls.
Jia et al. (2022)	22 NPMU 34 MAP 33 Schizophrenia (SCZ) 53 HCs	Structural MRI	Medial frontal gyrus Middle frontal gyrus Inferior frontal gyrus Gyrus rectus Precuneus	MAP and SCZ: similar grey matter reductions in the frontal cortex, particularly in prefrontal areas vs. HCs.
Blake et al. (2025)	27 MAP 36 SCZ 32 HCs	Structural MRI	Hippocampus Caudate Nucleus accumbens (NAcc) Putamen Globus pallidus Amygdala	SCZ/MAP: smaller left amygdala and thinner frontal cortex. SCZ: larger caudate, putamen, and NAcc vs. HCs SCZ: larger right globus pallidus and NAcc vs. MAP
Delvecchio et al. (2020)	10 cannabis-induced psychosis (CIP) 12 Non-psychotic cannabis users (NPCU)	Structural MRI	Superior frontal gyrus (SFG) Superior temporal gyrus (STG) Precentral gyrus Insula Precuneus Medial occipital gyrus (MOG) Fusiform Hippocampus	CIP: extensive grey matter decreases in right SFG, right precentral, right STG, insula bilaterally, right precuneus, right MOG, right fusiform gyrus, and left hippocampus
Ghosh et al. (2022)	31 SZC 28 CIP 30 HCs	Structural MRI	Cortical thickness	Lowest cortical thickness in the SZC, followed by CIP and the HCs SZC: Reduced cortical thickness than HCs in the middle and inferior frontal, right entorhinal, and left postcentral regions SZC: Lower than CIP in bilateral postcentral and right middle frontal regions
Rapp et al. (2013)	37 At-risk mental state (ARMS) 23 First episode psychosis (FEP)	Structural MRI	Cingulum	Negative associations of current cannabis use with grey matter volume of the cingulate cortex
Batalla et al. (2018)	30 chronic cannabis users 29 HCs	Structural MRI	Hippocampus Subregions of hippocampus	Reductions in hippocampal volume in heavy cannabis users compared to non-users Larger subregion volumes in cannabis users (CA1, CA2, CA3, CA4)
Penzel et al. (2021)	102 CIP	Structural MRI	Cortical grey matter volumes	Increased grey matter volume (GMV) in cerebellar network linked to early cannabis use Larger cerebellar volume associated with reduced GMV in insula, STG, and IFG.
Buchy et al. (2016)	132 Clinical high risk (CHR) cannabis users 387 CHR non-users 204 HCs	Structural MRI	Thalamus Hippocampus Amygdala	CHR users: significantly smaller amygdala than CHR non-users
Willi et al. (2016)	74 cocaine dependent nonpsychotic individuals 29 individuals with cocaine-associated psychosis	Structural MRI	Thalamus Hippocampus Caudate	Cocaine-associated psychosis: smaller thalamus and left hippocampus volumes
Alexander et al. (2019)	39 Stimulant-Induced Psychosis (dependent) 18 Schizophrenia (non-dependent) 39 Schizophrenia (dependent)	Structural MRI Diffusion tensor imaging	Temporal gyrus Frontal gyrus Hippocampus Amygdala Parietal operculum Anterior insula	Schizophrenia non-dependent group: larger left planum temporale and parietal operculum volumes Reduced fractional anisotropy and increased radial diffusivity in patients with stimulant-induced psychosis

Despite the comprehensive search strategy employed in this systematic review, which included specific terms for opioids, ecstasy, and hallucinogens, no structural neuroimaging studies were identified that met the eligibility criteria for these substance categories. This absence of data highlights a significant gap in the current literature, confirming that structural neuroimaging research into substance-induced psychosis is largely confined to cannabis, methamphetamine and cocaine.

### Methamphetamine-Induced Psychosis

To our knowledge, the first structural brain imaging study on this subject was conducted by Aoki et al. [22]. The authors examined frontal and parietal lobe regions associated with moral judgment—areas extensively studied in schizophrenia—in patients with methamphetamine-induced psychosis. Using voxel-based morphometry, they compared structural magnetic resonance imaging (MRI) data from 20 patients with methamphetamine-induced psychosis and 20 age- and sex-matched healthy controls, controlling for intracranial volume. The study revealed significant grey matter volume reductions in several frontal lobe regions, particularly the dorsomedial, ventromedial, dorsolateral, and ventrolateral prefrontal cortices. Reductions were also detected in left perisylvian structures such as the inferior frontal gyrus and anterior superior temporal gyrus, as well as in the orbitofrontal region and associated white matter. Moreover, decreased grey matter volumes in the medial frontal cortex were significantly correlated with greater positive symptom severity. The perisylvian changes mirrored findings in schizophrenia, whereas orbitofrontal alterations were interpreted as reflecting antisocial traits [22].

Breen et al. conducted a smaller-scale investigation combining neuroimaging with genome-wide RNA-Seq blood transcriptome analysis [23]. The study included 10 methamphetamine-dependent individuals without psychosis, 10 with methamphetamine-induced psychosis, and 10 healthy controls. The researchers identified co-expressed gene modules associated with subcortical brain regions and psychometric features. One module, enriched with 61 genes involved in ubiquitin-mediated proteolysis downregulation, was related to the anterior corpus callosum and nucleus accumbens. Another, linked to psychoticism and circadian clock regulation, was associated with the corpus callosum. Machine learning analysis identified blood-based biomarkers capable of distinguishing methamphetamine users from healthy controls with 87% accuracy, and differentiating psychotic from non-psychotic users with 95% accuracy. Structural analyses revealed significantly smaller bilateral hippocampal volumes in the psychosis group. These findings suggest that methamphetamine-induced psychosis may share molecular and neurocognitive underpinnings with schizophrenia and could serve as a model for identifying early biomarkers [23].

Uhlmann et al. further compared cortical thickness and subcortical volumes in 21 individuals with methamphetamine dependency, 19 patients with methamphetamine-induced psychosis, and 19 healthy controls [24]. MRI data demonstrated significant cortical thinning in the orbitofrontal region, inferior frontal gyrus, insula, fusiform gyrus, and inferior temporal gyrus in the psychosis group relative to non-psychotic individuals who use methamphetamine. Smaller hippocampal volumes were also observed in patients with methamphetamine-induced psychosis compared with both control groups. Emotional regulation difficulties were common across individuals who use methamphetamine but were specifically linked to cortical thinning in the inferior temporal gyrus, inferior frontal gyrus, and orbitofrontal cortex in psychotic cases. These findings suggest that methamphetamine-induced psychosis is characterised by cortical and hippocampal alterations similar to those in primary psychotic disorders [24].

Orikabe et al. focused on medial temporal lobe structures, performing MRI scans in 20 patients with methamphetamine-induced psychosis and 20 matched healthy controls [25]. They observed bilateral reductions in hippocampal and amygdalar volumes, with the amygdala showing a more pronounced decrease. Total grey matter, white matter, brain, and intracranial volumes were also reduced in the psychosis group. After controlling for these variables, the volumetric reductions in the hippocampus and amygdala remained significant. The authors concluded that this pattern—particularly the pronounced amygdalar reduction—may represent a distinctive morphometric feature of methamphetamine-induced psychosis [25].

Jia et al. compared structural brain changes among 22 individuals who use methamphetamine without psychosis, 34 with methamphetamine-induced psychosis, 33 patients with schizophrenia, and 53 healthy controls [26]. Significant grey matter reductions were identified in the frontal cortex, especially prefrontal areas, in both schizophrenia and methamphetamine-induced psychosis, with more severe reductions in schizophrenia. Notably, longer abstinence durations were associated with partial volumetric recovery in methamphetamine-induced psychosis, though this did not coincide with full symptom remission. Furthermore, longer psychotic phases were correlated with greater regional volume reductions, indicating a dose–response relationship between illness duration and structural change. The study concluded that methamphetamine-induced psychosis is associated with frontal grey matter loss similar to schizophrenia, potentially mediated by neuropathological mechanisms linked to symptom fluctuation during withdrawal [26].

In a recent study, Blake et al. investigated cortical thickness, subcortical morphology, and pro-inflammatory cytokines in 27 patients with methamphetamine-induced psychosis, 36 patient diagnosed with schizophrenia, and 32 healthy controls [27]. Neuroimaging revealed significantly reduced left amygdala volumes and frontal cortical thickness in both psychosis groups compared with healthy controls. Additionally, patients diagnosed with schizophrenia showed larger caudate nucleus, putamen, and nucleus accumbens volumes compared with healthy controls and increased right globus pallidus and nucleus accumbens volumes relative to the methamphetamine-induced psychosis group. No significant intergroup differences were observed in pro-inflammatory cytokine levels or their associations with neuroimaging findings. The authors concluded that the observed volumetric increases in the globus pallidus and nucleus accumbens among patients diagnosed with schizophrenia, compared to those with methamphetamine-induced psychosis, suggest that psychotic symptoms in these conditions may arise from distinct neurobiological mechanisms [27].

### Cannabis-Induced Psychosis

Delvecchio et al. investigated structural brain alterations in individuals with long-term cannabis use who developed psychosis compared with individuals with long-term use without psychosis [28]. MRI analyses revealed widespread grey matter changes in the bilateral insula, right precentral gyrus, superior frontal gyrus, right superior temporal gyrus, right precuneus, right medial occipital gyrus, right fusiform gyrus, and left hippocampus in patients with cannabis-induced psychosis. A significant negative correlation was found between the Brief Psychiatric Rating Scale (BPRS) Activity subscale and the volume of several affected regions. These findings suggest that specific grey matter volume reductions in cannabis-induced psychosis may underpin the emergence of psychotic symptoms [28].

In a study by Ghosh et al., cortical thickness and brain surface parameters were compared among patients diagnosed with schizophrenia and heavy cannabis use, patients with cannabis-induced psychosis, and healthy controls [29]. Analysis revealed significant group differences in cortical thickness, cortical depth, and gyrification. Reductions in these neurodegenerative markers were most pronounced in patients diagnosed with schizophrenia with heavy cannabis use, followed by those with cannabis-induced psychosis, relative to healthy controls. These alterations were most evident in frontal regions, with more limited changes in the temporal and parietal cortices. Specifically, cortical thickness was significantly reduced in the right entorhinal, middle, and inferior frontal, and left postcentral regions in patients diagnosed with schizophrenia with heavy cannabis use compared with healthy controls, and in the bilateral postcentral and right middle frontal regions compared with patients with cannabis-induced psychosis. Moreover, a negative correlation was observed between the duration of cannabis use and parietal and occipital cortical thickness in both psychosis groups. Overall, the researchers demonstrated that cortical abnormalities are present in both schizophrenia and cannabis-induced psychosis, but are more pronounced in schizophrenia, supporting the notion that these disorders may involve distinct neurobiological mechanisms [29].

In another cannabis-related study, Rapp et al. examined cingulum volumes for the first time in patients with first-episode psychosis and individuals at risk for psychosis [30]. This cross-sectional study included 23 patients with first-episode psychosis and 37 participants with an at-risk mental state, whose cingulum volumes were assessed using MRI and manual tracing. Repeated-measures analyses of covariance were conducted to determine the association between current cannabis use and cingulum volume, controlling for sex, total brain volume, alcohol consumption, and antipsychotic use. The researchers identified a three-way interaction involving anterior and posterior cingulum regions, hemispheric lateralisation, and cannabis use. Subsequent analyses revealed a significant negative effect of cannabis use on posterior cingulum volume, independent of hemisphere, diagnostic group, or other covariates. A similar negative effect was observed in the left anterior cingulum, regardless of diagnosis. Furthermore, in patient groups, greater cannabis use was negatively correlated with grey matter volume in the cingulate cortex, a region rich in cannabinoid type 1 receptors. This relationship was absent in healthy controls, suggesting that individuals with first-episode psychosis or at-risk mental states may be particularly sensitive to CB1 receptor stimulation from exogenous cannabis [30].

Batalla et al. combined neuroimaging and genetic analyses to investigate the effects of heavy cannabis use on brain structure [31]. Although not conducted in patients with substance-induced psychosis, the study was included for its relevance in illustrating cannabis-related structural brain changes and their potential link to psychotic symptoms. The researchers examined associations between catechol-O-methyltransferase (COMT), dopamine transporter (DAT1), and brain-derived neurotrophic factor (BDNF) gene polymorphisms and total and subregional hippocampal volumes. The findings indicated that both cannabis use and genetic polymorphisms independently influenced hippocampal morphology. Significant associations were observed between cannabis use and reductions in total and regional hippocampal volumes, with total hippocampal and fissure subregion volumes particularly affected by cannabis use and DAT1 variation. They concluded that cannabis use may alter the typical relationship between DAT1 polymorphism and hippocampal structure, suggesting gene-specific and region-specific effects of cannabis on hippocampal integrity [31].

In a multicentre study, Penzel et al. investigated the detrimental effects of cannabis use on brain maturation [32]. The sample included 102 individuals who use cannabis who had recently developed psychosis, and analyses focused on the relationship between age at cannabis initiation and psychosis development. Using source-based morphometry with spatial constraints, the researchers examined structural brain networks previously shown to be altered in schizophrenia. They found that an earlier age of cannabis initiation was associated with more severe positive psychotic symptoms. Moreover, increased grey matter volume in a cerebellar network—previously identified as abnormal in schizophrenia—was significantly linked to early cannabis use. This increase was also inversely associated with grey matter volume in another network encompassing the insula, superior temporal gyrus, and inferior frontal gyrus, regions likewise implicated in schizophrenia. Overall, they identified structural brain alterations resembling those observed in schizophrenia and suggested that early cannabis use may disrupt normal brain network maturation, thereby increasing vulnerability to psychosis later in life [32].

In a study investigating substance use and psychosis risk, Buchy et al. examined subcortical brain structures in individuals at high risk for psychosis [33]. The sample included 132 cannabis-using high-risk participants, 387 non-using high-risk participants, and 204 healthy non-using controls. MRI analyses focused on thalamic, hippocampal, and amygdalar volumes. When all high-risk individuals were compared with healthy controls, no significant differences were observed in any subcortical region. However, within the high-risk group, individuals who use cannabis showed significantly larger amygdala volumes than non-users, while hippocampal and thalamic volumes did not differ. When analyses were restricted to participants without severe alcohol use, smaller hippocampal volumes were identified in individuals who use cannabis, and the amygdala differences disappeared. After adjusting for alcohol and cigarette use, all group differences lost statistical significance. They highlighted that these findings underscore the need to account for confounding effects of other substances when examining the relationship between cannabis use and psychosis risk [33].

### Cocaine- and Other Stimulant-Induced Psychosis

Cocaine, a psychostimulant, can induce psychosis that closely resembles schizophrenia, making differentiation challenging. It has therefore been proposed that shared neurobiological mechanisms and neural substrates may underlie both conditions [34].

In their study, Willi et al. examined subcortical grey matter using voxel-based morphometry, comparing 67 individuals with cocaine dependency and no psychosis with 29 individuals with cocaine-induced psychosis [34]. They found significantly smaller thalamic and left hippocampal volumes in the cocaine-induced psychosis group, even after controlling for age, total brain volume, and other substance use. No significant differences were observed in caudate nucleus volume between the two groups. The authors concluded that hippocampal and thalamic alterations in cocaine-induced psychosis parallel those seen in schizophrenia, suggesting that such structural changes may be associated with the emergence of psychosis more generally, rather than being specific to schizophrenia [34].

In a comparative study, Alexander et al. examined neurocognitive, clinical, and neuroanatomical differences among patients with stimulant-induced psychosis, patients with schizophrenia with psychostimulant dependence, and those with schizophrenia without such dependence [35]. No significant group differences were found in neurocognitive performance. However, neuroimaging revealed notable structural variations. Specifically, patients with schizophrenia and psychostimulant dependence had significantly smaller left temporal and parietal lobes than those without. DTI analyses further showed reduced fractional anisotropy and increased radial diffusivity in patients with stimulant-induced psychosis compared with schizophrenia patients with psychostimulant dependence. The authors concluded that while schizophrenia and stimulant-induced psychosis share certain structural brain alterations, distinct neuroanatomical patterns are also evident. Moreover, psychostimulant dependence in schizophrenia may be associated with unique neurobiological substrates [35].

### Summary and Conclusion

When evaluated collectively, structural neuroimaging studies of substance-related psychosis reveal neuroanatomical patterns that largely resemble those observed in primary psychotic disorders such as schizophrenia. The presence of shared features, including cortical thinning, reduced frontal grey matter and subcortical volumetric loss in regions such as the hippocampus and thalamus, suggests the existence of a ‘final common pathway’. In this pathway, various factors — including exogenous neurotoxicity from substances, genetic variations and developmental timing — converge to produce the structural signatures of psychosis. Although the specific regions affected may differ depending on the substance, the overlap in frontal and subcortical deficits indicates a shared neurobiological mechanism underlying these diverse triggers.

Despite these similarities, our review highlights significant substance-specific variations that suggest partially divergent neuropathological mechanisms. For instance: Methamphetamine-induced psychosis is characterised by more pronounced reductions in the amygdala compared to primary psychosis, and distinct differences in the volumes of the globus pallidus and the nucleus accumbens. Cannabis-induced psychosis has been shown to involve the cerebellum in a unique manner and to elicit specific sensitivity in the cingulate cortex, particularly when use commences during the early stages of brain maturation. Overall, structural deficits in schizophrenia are more pervasive and widespread than those observed in substance-induced cases, suggesting that primary psychosis is characterised by a more global disruption of neurodevelopmental integrity. This pattern is consistent with diffuse alterations across multiple brain regions. By contrast, substance-induced states more commonly reflect region-specific neurotoxic effects, indicating a comparatively localised pattern of structural involvement.

## Future Directions

**Fig. 1:**
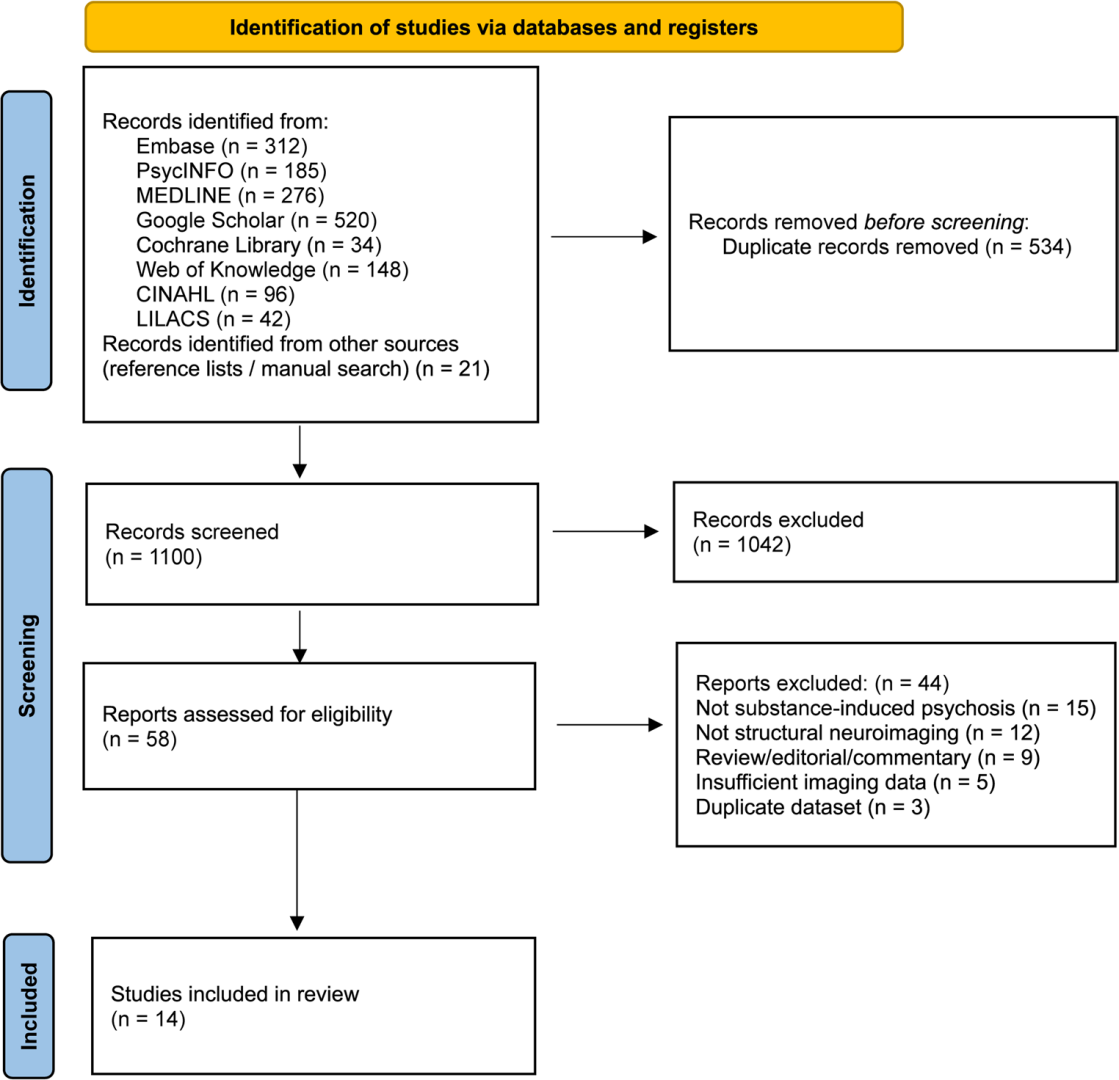
PRISMA flow diagram illustrating the study selection process, from initial database identification to the final inclusion of 14 studies

To advance the field, future research should prioritise longitudinal, multimodal designs to clarify the relationship between substance use and structural brain changes. Identifying reliable biomarkers is crucial for improving diagnostic precision in the acute phase, particularly when symptoms persist beyond the first month of abstinence. Such approaches would also enhance prognostic accuracy by distinguishing irreversible neuroanatomical damage from the potential for partial volumetric recovery, as reported in some methamphetamine-using populations. Moreover, defining disorder-specific neurobiological signatures—such as cerebellar network disruptions linked to early-onset cannabis use—may inform the development of targeted, personalised pharmacological and neurorehabilitative interventions. However, significant barriers remain, primarily the substantial methodological heterogeneity in neuroimaging techniques and analysis software that complicates cross-study comparisons. Researchers must also contend with the high prevalence of polydrug use, which makes isolating the structural impact of a single substance a formidable statistical and recruitment challenge. These efforts are further complicated by the inherent diagnostic overlap between substance-induced psychosis and primary psychotic disorders; because substance use can frequently accelerate the course of an underlying condition, it remains biologically difficult to “cleanly” separate these categories. Finally, the clinical instability and ongoing substance use often observed in patients during acute psychotic states lead to high rates of loss-to-follow-up, making the necessary long-term data collection exceptionally difficult to maintain (Fig. 1).

## Limitations

The most prominent limitation of the current manuscript is the narrow scope of the available literature. While we conducted a comprehensive search, the evidence base is effectively restricted to three major substances. This concentration reflects a significant gap in the psychiatric field; without structural data for other illicit substances, we cannot yet determine if the “final common pathway” described here is truly universal across all substance-induced states. Furthermore, methodological heterogeneity remains a major hurdle. The use of different analysis software and imaging modalities (such as manual tracing versus automated segmentation) introduces a level of variance that can obscure subtle regional changes. This is compounded by the fact that most participants in these studies are polydrug users. While some researchers control for alcohol or tobacco use, the synergistic effect of multiple substances on brain morphology is rarely fully accounted for, potentially masking substance-specific neurotoxicity. Finally, we must acknowledge the “diagnostic moving target” inherent in this population. Because nearly half of individuals with cannabis-induced psychosis may later receive a schizophrenia diagnosis, it is difficult to ascertain whether the structural alterations identified in our review are consequences of the substance itself or early biomarkers of an underlying primary disorder that was simply unmasked by substance use. Future studies utilising longitudinal designs with first-episode patients are essential to untangle these complex causal relationships.

## Key References

- Blake L, Williams KC, Uhlmann AA, Temmingh H, Burger A, Stein DJ, et al. Subcortical volumes, frontal cortical thickness, and pro-inflammatory cytokines in schizophrenia versus methamphetamine-induced psychosis. Brain Imaging Behav. 2025;19(4):874–888.
  - ○ This study is critical as it directly compares methamphetamine-induced psychosis with schizophrenia, suggesting that while both groups show frontal thinning and reduced amygdala volume, the distinct volumetric differences in the globus pallidus and nucleus accumbens point toward different underlying neurobiological mechanisms.
- Jia X, Wang J, Jiang W, Kong Z, Deng H, Lai W, et al. Common gray matter loss in the frontal cortex in patients with methamphetamine-associated psychosis and schizophrenia. Neuroimage Clin. 2022;36:103259.
  - ○ This research highlights the commonality of frontal grey matter loss between schizophrenia and methamphetamine-induced psychosis, while also demonstrating that longer abstinence leads to partial volumetric recovery, providing important insights into the potential reversibility of these structural changes.
- Ghosh A, Kaur S, Shah R, Oomer F, Avasthi A, Ahuja CK, et al. Surface-based brain morphometry in schizophrenia vs. cannabis-induced psychosis: A controlled comparison. J Psychiatr Res. 2022;155:286–294.
  - ○ By comparing cannabis-induced psychosis and schizophrenia through surface-based morphometry, this study reveals that while both disorders involve frontal cortical abnormalities, the deficits are significantly more pronounced in schizophrenia, helping to delineate the severity gap between primary and substance-induced states.
- Penzel N, Antonucci LA, Betz LT, Sanfelici R, Weiske J, Pogarell O, et al. Association between age of cannabis initiation and gray matter covariance networks in recent onset psychosis. Neuropsychopharmacology. 2021;46(8):1484–1493.
  - ○ This reference is essential for understanding the developmental impact of substance use, as it demonstrates that earlier cannabis initiation disrupts brain network maturation—specifically in cerebellar and insular networks—thereby increasing vulnerability to psychosis.

## Data Availability

No datasets were generated or analysed during the current study.
